# When intestinal ulceration meets hematologic malignancies: clinical features and mortality from a pooled individual-patient data systematic review

**DOI:** 10.3389/fimmu.2026.1808470

**Published:** 2026-05-29

**Authors:** Song Su, He Zhou, Zimeng Wang, Yanting Shi, Xiaofei Li, Longsong Li, Yawei Bi, Jie Liang, Ningli Chai

**Affiliations:** 1 Senior Department of Gastroenterology, Chinese PLA General Hospital, Beijing, China; 2 State Key Laboratory of Cancer Biology and National Clinical Research Center for Digestive Diseases, Xijing Hospital of Digestive Diseases, Fourth Military Medical University (Air Force Medical University), Xi’an, China; 3 Second Division of Cadre Ward, General Hospital of Central Theater Command, Wuhan, China

**Keywords:** Beçhet’s disease, inflammatory bowel disease, intestinal ulcers, myelodysplastic syndromes, aplastic anemia, pancytopenia

## Abstract

**Background and aims:**

Intestinal ulceration with pancytopenia is rare but associated with high mortality, reported mostly in small case series. It often occurs in patients with inflammatory bowel disease (IBD) or intestinal Behçet’s disease (BD) and concurrent myelodysplastic syndrome (MDS) or aplastic anemia (AA). We performed a systematic review and pooled individual−patient data analysis to characterize the clinical features, evaluate mortality, and explore prognostic factors.

**Methods:**

We searched PubMed, Embase, ClinicalTrials.gov, and PROSPERO from database inception to November 10, 2025, for eligible case reports, case series, and cohort studies. Individual patient data were extracted and pooled to describe clinical features and evaluate mortality. Cox proportional hazards models were used to assess factors associated with mortality.

**Results:**

Sixty studies (128 patients) were included. In patients with BD and MDS, a marked female predominance (68.9%) and a high prevalence of trisomy 8 (85.7%) were observed, with ulcers predominantly located in the ileocecal region. In IBD-associated cases, patients with pancytopenia were characterized by a strong male preponderance (up to 90%) and rare trisomy 8 abnormalities; intestinal involvement typically manifested as ileal ulcers in Crohn’s disease (CD) and pancolitis in ulcerative colitis (UC). Diagnoses were frequently concurrent, or intestinal symptoms preceded the onset of hematologic disease. Overall mortality was substantial, ranging from 31.3% to 44.4%. In subgroup analyses, male sex and MDS evolution were associated with higher mortality in patients with BD and MDS, while high-risk MDS subtypes were linked to poorer survival in those with IBD and MDS.

**Conclusion:**

The coexistence of intestinal ulcers and pancytopenia is associated with substantial mortality, reaching as high as 44.4%. The observed sex distribution and trisomy 8 patterns should be interpreted as hypothesis-generating clinical clues rather than definitive evidence of pathogenic significance or diagnostic utility. By systematically characterizing the clinical features and prognosis of this rare condition, this study provides a useful synthesis of the available evidence and highlights priorities for future prospective and mechanistic studies.

**Systematic Review Registration:**

https://www.crd.york.ac.uk/prospero/display_record.php?ID=CRD42020165205, identifier CRD42020165205.

## 1 Introduction

Intestinal ulcers are a common finding on lower gastrointestinal endoscopy and may involve any segment of the small or large intestine. The differential diagnosis is extensive, including inflammatory bowel disease (IBD), Behçet’s disease (BD), lymphoma, intestinal infections, neoplasms, vascular abnormalities, ischemic injury, radiation injury, and tuberculosis (1). While the majority of lesions are benign, a distinct subset of patients presents with concurrent pancytopenia, creating a more complex clinical picture with diagnostic and therapeutic challenges.

Pancytopenia, defined as concurrent reductions in white blood cells, red blood cells, and platelets, may reflect bone marrow failure, peripheral destruction or sequestration, or treatment-related toxicity. In patients with IBD or BD who develop intestinal ulcers together with unexplained cytopenias, myelodysplastic syndromes (MDS) and aplastic anemia (AA) are particularly relevant hematologic conditions because they represent the most frequently reported marrow disorders in this clinical setting (2, 3). Among them, MDS deserves particular attention because it accounts for most published cases of intestinal ulceration with pancytopenia and may be associated with immune dysregulation and cytogenetic abnormalities.

MDS is a clonal hematopoietic stem-cell disorder characterized by ineffective hematopoiesis, persistent cytopenias, and risk of progression to acute myeloid leukemia; some patients also develop autoimmune or autoinflammatory manifestations. At the molecular level, disrupted hematopoietic stem-cell homeostasis, including epigenetic dysregulation and cellular stress responses, together with cytogenetic abnormalities, may contribute to both marrow failure and inflammatory manifestations (4, 5). Among these abnormalities, trisomy 8 is of particular interest because it has been repeatedly associated with intestinal Behçet-like manifestations and may represent a potential biological link between hematologic disease and intestinal ulceration.

When intestinal ulcers and pancytopenia coexist, clinical management becomes particularly challenging. In our previous study (6), we noted a rapid disease course and poor treatment responses. Prior case reports and small series have also described refractory intestinal ulceration and severe outcomes in patients with coexisting intestinal disease and MDS/AA, supporting the need for systematic synthesis (7–10). Major gastroenterology guidelines (e.g., ECCO, BSG, AGA) and the Toronto consensus provide no specific recommendations for this scenario. Our preliminary literature search indicated that the most common gastrointestinal diagnoses are IBD or BD, and the most frequent hematologic diagnoses are AA or MDS. However, evidence is largely limited to case reports and small series, affected patients appear to share distinctive clinical characteristics, and mortality appears high. To address these gaps, we conducted a systematic review of patients with IBD or intestinal BD combined with MDS or AA, the most common combinations, to delineate their clinical characteristics and outcomes.

## 2 Methods

This systematic review is reported in accordance with the Preferred Reporting Items for Systematic Reviews and Meta-analyses (PRISMA) Statement (11) and was registered in PROSPERO (CRD42020165205).

### 2.1 Data sources and searches

We performed a comprehensive literature search of PubMed, EMBASE, ClinicalTrials.gov, and PROSPERO from database inception to November 10, 2025. ClinicalTrials.gov was also reviewed for ongoing or recently completed trials, and PROSPERO for ongoing or recently completed systematic reviews. The detailed search strategies are available in the Supplemental Material (Search Strategy). No language restrictions were applied at the search stage; however, only English-language full texts were eligible during screening. This restriction was applied to ensure accurate extraction of granular case-level data from full-text reports, although it may have introduced language bias. We additionally hand-searched the reference lists of included publications and consulted an expert (JL) in this field.

### 2.2 Study selection

Studies were selected using an iterative process. Two independent investigators (SS and HZ) reviewed study titles and abstracts, and studies that satisfied the inclusion criteria were retrieved for full-text assessment. Disagreements were resolved by a third investigator (JL). Studies reporting at least one case of IBD/BD combined with MDS/AA were considered eligible for this systematic review. Both adult and pediatric patients from any country were eligible. Studies published in languages other than English were excluded. Exclusion criteria included review articles, conference abstracts, editorials, and letters without original cases, as well as irrelevant studies. Reports of intestinal ulcers without a definitive diagnosis were excluded (7). When two studies involved overlapping populations, the most comprehensive and most recently published study was included.

### 2.3 Data extraction and quality assessment

The following data were extracted from each included study using a standardized data-collection form: first author, publication year, country where the study was conducted, number of reported cases, gender, age (years) at IBD/BD/MDS/AA diagnosis, the interval (years) between IBD/BD and MDS/AA diagnoses, IBD type, BD subtype, intestinal extent, trisomy 8, treatments, follow-up duration (years), MDS evolution and vital status. Risk of bias was assessed using the Joanna Briggs Institute (JBI) Critical Appraisal Tools (12). The JBI tools were applied according to study design to appraise reporting and methodological quality, but these ratings do not eliminate the inherent limitations of case reports and case series. Two researchers (SS and HZ) independently appraised the studies; disagreements were resolved by a third investigator (JL).

### 2.4 Outcomes assessment and definitions

The primary outcome was the description of clinical characteristics, including sex distribution, the temporal relationship between intestinal ulceration and pancytopenia, age at diagnosis of each disease, intestinal extent, MDS subtype, and the frequencies of trisomy 8 and MDS evolution. Secondary outcomes were overall mortality among patients with IBD/BD with concurrent MDS/AA and risk factors for mortality among patients with IBD/BD and MDS. The diagnosis interval was defined as the time between the diagnoses of the two conditions. Follow−up was measured from the time both conditions had been diagnosed to death or the end of follow−up. We evaluated the impact of MDS subtype on mortality by stratifying patients as low risk (RA, RCMD, RARS) versus high risk (all other subtypes).

### 2.5 Data synthesis and analysis

Data were summarized as proportions, means, or medians, as appropriate. In case series studies, data were extracted comprehensively but analyzed at the individual-patient level. Because patient-level data were extracted from published reports rather than obtained as original datasets from investigators, this study should be considered a systematic review with pooled analysis of published case-level data rather than a conventional investigator-led IPD meta-analysis. Given the anticipated clinical and methodological heterogeneity across case reports and case series, we did not perform study-level effect meta-analysis; instead, we summarized the published case-level data descriptively and stratified analyses by disease combination. Missing data were not imputed; analyses were performed using available cases, with denominators reported where applicable. All analyses were performed using IBM SPSS Statistics, version 21. Descriptive statistics were performed on data extracted from all included studies. Mann-Whitney U test, χ² test, or Fisher’s exact test were used to compare between survivors and non-survivors where appropriate. We planned univariable and multivariable Cox proportional hazards regression, but owing to an insufficient number of deaths and to avoid overfitting, we conducted only univariable analyses. A two-sided P value <0.05 was considered statistically significant.

## 3 Results

### 3.1 Characteristics and quality of included studies

**Figure 1:**
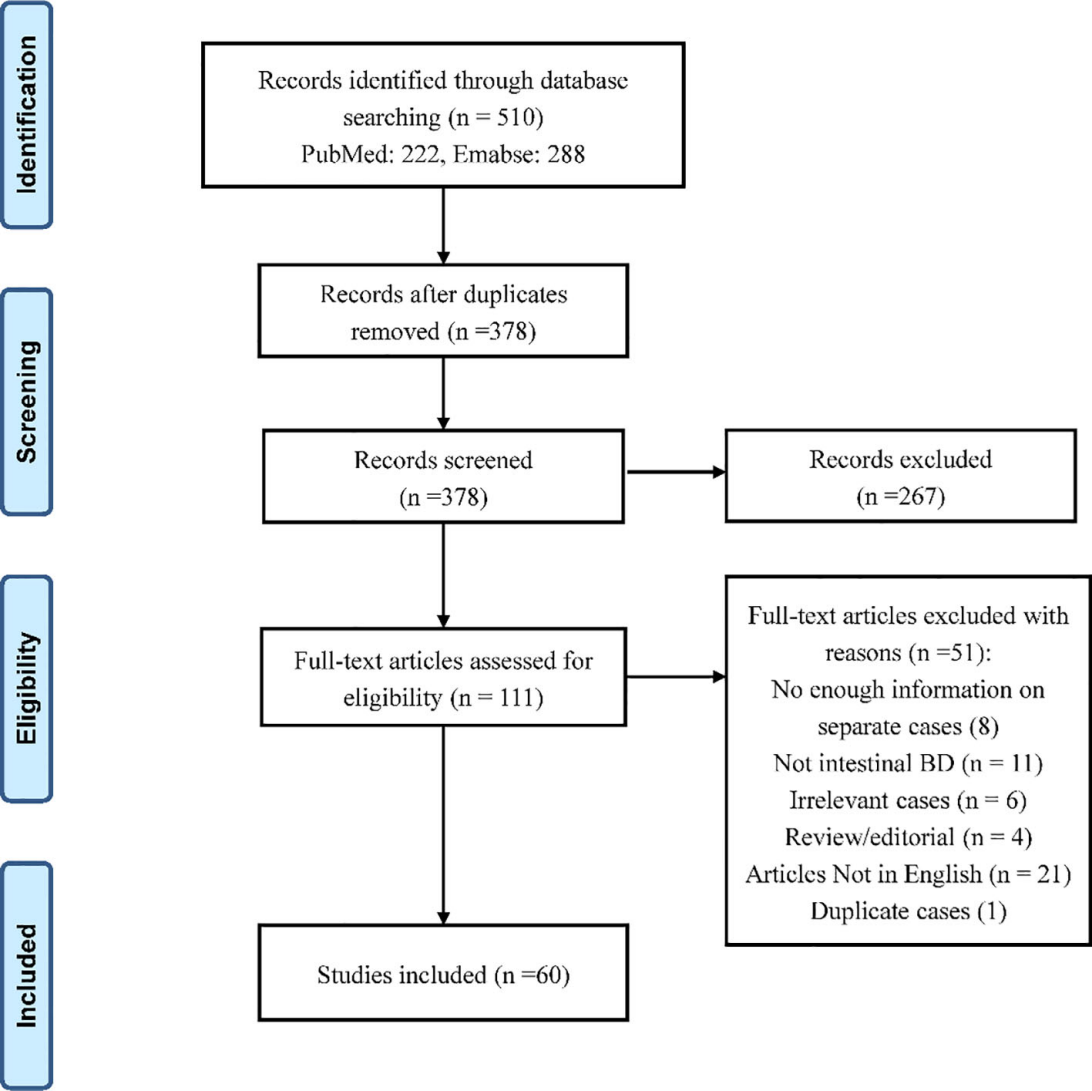
Flow chart for literature search and inclusion.

The PRISMA flow diagram (**Figure 1**) outlines the results of studies identification, screening, eligibility assessment, and inclusion. The database search initially yielded 510 records. After removal of duplicates and screening of titles and abstracts, 111 full-text articles were reviewed; ultimately, 60 studies (3, 6–10, 13–66) involving 128 patients met the eligibility criteria. Baseline characteristics are presented in **Supplementary Tables 1–3**, stratified by BD & MDS, IBD & MDS, and IBD & AA. Individual patient data (IPD) are provided in Supplemental Data 1–3. Most included papers were case reports (n=42; 70.0%), with 18 case series (30.0%). By subgroup, 28 studies reported BD & MDS (n=45; 35.2%), 19 reported IBD & MDS (n=62; 48.4%), 13 reported IBD & AA (n=18; 14.1%), and one Korean study reported BD & AA (n=3; 2.3%). Studies of BD & MDS were predominantly conducted in Japan, whereas IBD & MDS reports were mainly from the USA (**Table 1**). Study quality is summarized in **Supplementary Figures 1**–**3**, with most studies meeting a majority of applicable JBI appraisal items; however, these results should be interpreted cautiously because most included studies were case reports or case series. Unless otherwise specified, percentages are calculated among patients with available data, and denominators may vary due to missingness. For key variables, vital status was available in 109 of 128 patients, and trisomy 8 status was available in 78 patients with MDS.

**Table 1:** Summary of the characteristics of patients with concurrent benign intestinal ulcers and pancytopenia.

Characteristics	BD & MDS (n=45)	IBD & MDS (n=62)		IBD & AA (n=18)	
		CD & MDS (n=46)	UC & MDS (n=16)	CD & AA (n=10)	UC & AA (n=8)
Country, n (%)	Japan, 32/45 (71.1%)	USA, 20/46 (43.5%)	USA, 12/16 (75%)	China, 6/10 (60%)	UK, 2/8 (25%)
	Korea, 9/45 (20%)	China, 9/46 (19.6%)	China, 2/16 (12.5%)	USA, 2/10 (20%)	Japan, 2/8 (25%)
	Others, 4/45 (8.9%)	Belgium, 6/46 (13.0%)	UK, 2/16 (12.5%)	Others, 2/10 (20%)	Others, 4/8 (50%)
Male, n (%)	14/45 (31.1%)	34/46 (73.9%)	8/16 (50%)	9/10 (90%)	7/8 (87.5%)
Age at BD/IBD diagnosis (yrs)					
Mean (SD)	38.8 (22.5)	53.9 (19.6)	48.5 (17.2)	42.0 (16.0)	31.5 (19.8)
Median (range)	36.5 (4, 81)	56.0 (8, 87)	52.0 (18, 76)	41 (25, 74)	25 (9, 71)
Age at MDS/AA diagnosis (yrs)					
Mean (SD)	39.5 (21.3)	57.3 (19.0)	61.8 (16.3)	42.0 (18.3)	34.3 (20.0)
Median (range)	37.0 (4,81)	62.5 (9, 83)	68 (22, 76)	41.5 (14, 74)	32 (9, 72)
Diagnosis sequence, n (%)					
Simultaneously	21/43 (48.8%)	21/43 (48.8%)	2/15(13.3%)	5/9 (55.6%)	2/8 (25%)
IBD/BD early	15/43 (34.9%)	15/43 (34.9%)	13/15 (86.7%)	2/9 (22.2%)	5/8 (62.5%)
MDS/AA early	7/43 (16.3%)	7/43 (16.3%)	0/15 (0%)	2/9 (22.3%)	1/8 (12.5%)
Interval between two diseases (yrs), median (range)	4 (1,14)	6 (1, 34)	9 (1, 40)	1.5 (1, 2)	1 (1, 2)
Intestinal extent, n (%)					
Ileal	6/28 (21.4%)	15/37 (40.5%)	NA	3/9 (33.3%)	NA
IC	17/28 (60.7%)	12/37 (32.4%)	NA	4/9 (44.4%)	NA
Colonic	9/28 (32.1%)	11/37 (29.7%)	1/13 (7.7%)	4/9 (44.4%)	3/3 (100%)
Whole colon	4/28 (14.3%)	2/37 (5.4%)	11/13 (84.6%)	1/9 (11.1%)	NA
Rectum	1/28 (3.6%)	4/37 (10.8%)	1/13 (7.7%)	NA	NA
MDS subtype (FAB/WHO classification) (first three of the most frequent)					
	RA, 22/43 (51.2%)	RAEB, 20/44 (45.4%)	RA, 7/16 (43.8%)	NA	NA
	RAEB, 9/43 (20.9%)	RA, 12/44 (27.3%)	RARS, 4/16 (25.0%)	NA	NA
	RCMD, 8/43 (18.6%)	RARS, 8/44 (18.2%)	RAEB, 3/16 (18.8%)	NA	NA
Trisomy 8, n (%)					
Positive	36/42, (85.7%)	5/28 (17.9%)	0/8 (0%)	NA	NA
Negative	6/42, (14.3%)	23/28 (82.1%)	8/8 (100%)	NA	NA
MDS evolution, n (%)					
AML	4/45 (8.9%)	6/46 (13.0%)	3/16 (18.8%)	NA	NA
RAEB	1/45 (2.2%)	NA	NA	NA	NA
CML	NA	2/46 (4.3%)	NA	NA	NA
RAEB-t	NA	1/46 (2.2%)	NA	NA	NA
Follow-up (yrs), median (range)	1 (0, 5)	1.5 (0.1, 10)	1 (0, 7)	2 (0, 15)	1.25 (0, 4)
Vital status, n (%)					
Dead	13/36 (36.1%)	13/40 (32.5%)	5/16 (31.3%)	4/9 (44.4%)	3/8 (37.5%)
Alive	23/36 (63.9%)	27/40 (67.5%)	11/16 (68.8%)	5/9 (55.6%)	5/8 (62.5%)

AA, aplastic anemia; AML, acute myeloid leukemia; BD, Beçhet’s disease; CD, Crohn’s disease; CML, chronic myelomonocytic leukemia; IBD, inflammatory bowel disease; IC, ileocecal region; MDS, myelodysplastic syndrome; RAEB, refractory anemia with excess of blasts; RAEB-t: refractory anemia with excess blasts in transformation; UC, ulcerative colitis.

### 3.2 Clinical features of patients with intestinal ulcer and pancytopenia

**Figure 2:**
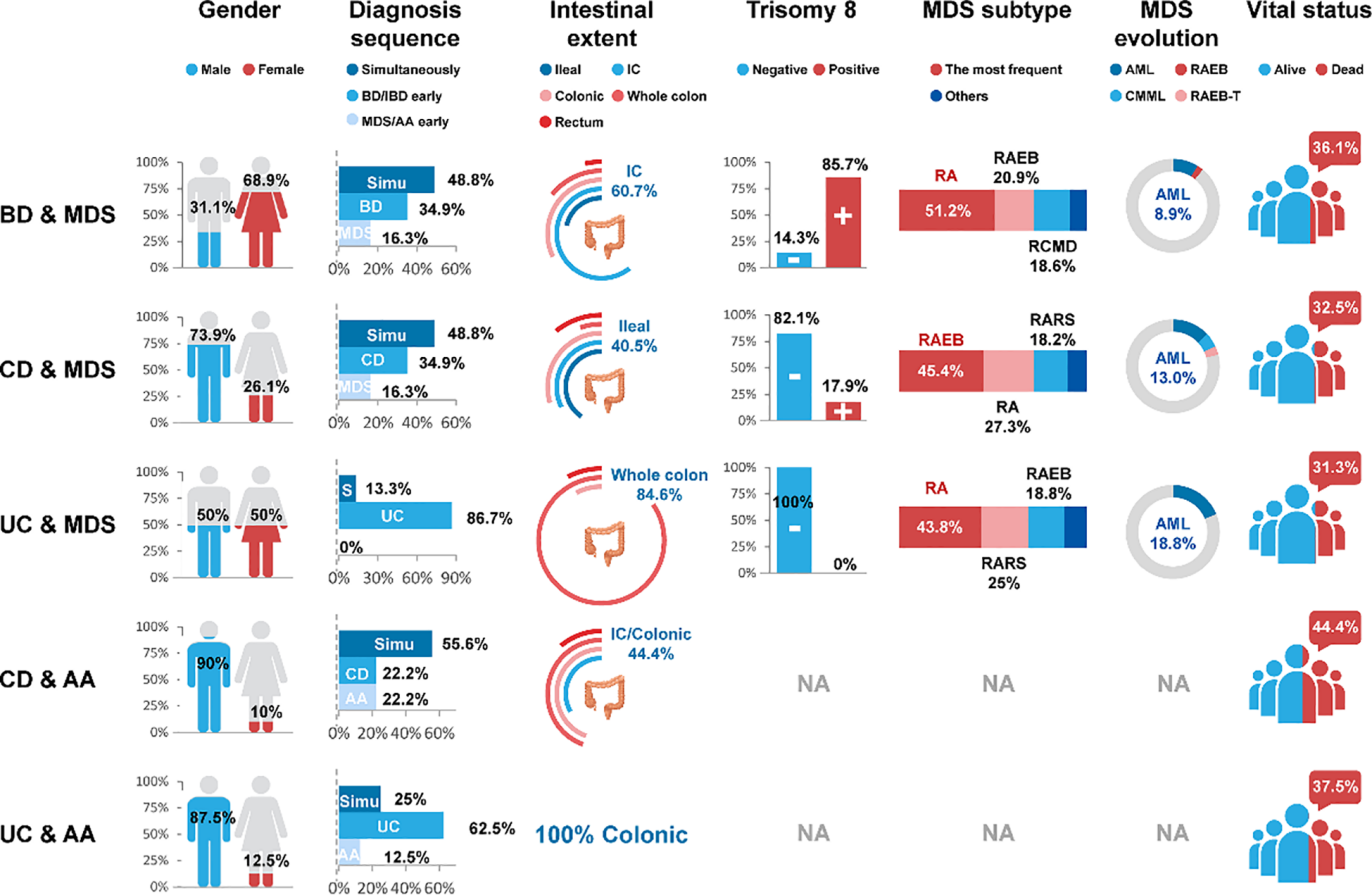
Schematic summary of clinical characteristics and mortality of patients with combined intestinal ulcers and pancytopenia.

The clinical features and mortality are summarized in **Table 1** and the schematic summary shown in **Figure 2**. A marked gender imbalance was observed: 68.9% of patients with BD & MDS were female, whereas most patients with IBD and pancytopenia were male (50-90%), with the highest proportion (90%) seen in those with CD and AA. In the majority of included patients, the two conditions were diagnosed either simultaneously or with IBD/BD preceding MDS/AA. The median age at BD/IBD diagnosis ranged from 25 to 56 years, and patients with CD & MDS had the highest median diagnostic age (56 years). The median age at MDS/AA diagnosis ranged from 32 to 68 with the highest age (68 years) observed in patients with UC & MDS. Furthermore, the median interval between the two diagnoses ranged from 1 to 9 years; patients with IBD & MDS had the longest median intervals: 6 years for CD & MDS and 9 years for UC & MDS.

Regarding the intestinal ulcer location, both the small and large intestine could be involved, but predominant sites differed by subgroup: ileocecal (IC) region in BD & MDS; ileal involvement in CD & MDS; whole colon involvement in UC & MDS; combined IC/colonic involvement in CD & AA, and purely colonic involvement in UC & AA. With respect to MDS subtypes, refractory anemia (RA) was the most frequent subtype in patients with UC & MDS (43.8%) and BD & MDS (51.2%), whereas most patients with CD & MDS (45.4%) had RAEB (refractory anemia with excess blasts). Of note, among 78 MDS patients tested for trisomy 8, the prevalence differed strikingly between BD-associated and IBD-associated cases: 85.7% of BD & MDS patients were trisomy 8 positive, compared with only 17.9% of CD & MDS patients; none of the tested UC & MDS patients had trisomy 8. Regarding MDS evolution, acute myeloid leukemia (AML) was the most common transformation, occurring in 8.9% of BD & MDS cases, 13.0% of CD & MDS cases, and 18.8% of UC & MDS cases.

### 3.3 Treatments and mortality

Survival outcomes were documented in 109 of the 128 included cases. The overall mortality was 34.9% (38/109), with 36.1% (13/36) in patients with BD & MDS, 32.5% (13/40) in CD & MDS, 31.3% (5/16) in UC & MDS, 44.4% (4/9) in CD & AA, and 37.5% (3/8) in UC & AA during a median follow-up of 2 years (range, 1–10 years) (**Table 1**). Comparisons of clinical characteristics between survivors and non-survivors are shown in **Table 2**. Apart from follow-up duration, only MDS subtype differed significantly between survivors and non-survivors with IBD & MDS, with high-risk MDS subtypes associated with higher mortality. For the following variables: gender, IBD type, diagnosis interval, diagnosis sequence, intestinal extent of ulcers, trisomy 8 and MDS evolution, there were no significant differences in mortality between survivors and non-survivors in any subgroup.

**Table 2:** Comparison of clinical characteristics between survival and non-survival patients.

Characteristics	BD & MDS			IBD & MDS			IBD & AA		
	Dead (n=13)	Alive (n=23)	p value	Dead (n=18)	Alive (n=38)	p value	Dead (n=7)	Alive (n=10)	p value
IBD type, n (%)						0.928			1
CD				13/18 (72.2%)	27/38 (71.1%)		4/7 (57.1%)	5/10 (50%)	
UC				5/18 (27.8%)	11/38 (28.9%)		3/7 (42.9%)	5/10 (50%)	
Gender, n (%)			0.153			0.115			1
Male	6/13 (46.2%)	5/23 (21.7%)		10/18 (55.6%)	29/38 (76.3%)		6/7 (85.7%)	9/10 (90%)	
Female	7/13 (53.8%)	18/23 (78.3%)		8/18 (44.4%)	9/38 (23.7%)		1/7 (14.3%)	1/10 (10%)	
Age at BD diagnosis (yrs)									
Mean (SD)	37.4 (24.8)	36.6 (24.0)		52.7 (18.7)	50.4 (19.3)		36.0 (20.4)	37.4 (18.4)	
Median (range)	36 (4, 81)	32 (4, 80)	0.838	56 (18, 82)	52 (8, 76)	0.777	28 (18, 71)	38 (9, 74)	NA
Age at MDS diagnosis (yrs)									
Mean (SD)	40.0 (23.5)	37.6 (22.9)		60.8 (18.2)	55.2 (18.6)		36.4 (21.5)	40.8 (18.8)	
Median (range)	37 (7, 81)	35 (4, 79)	0.682	66 (22, 82)	61 (9, 79)	0.219	29 (14, 72)	45 (9, 74)	NA
Diagnosis sequence, n (%)			0.074			0.712			1
Simultaneously	4/13 (30.8%)	13/22 (59.1%)		5/17 (29.4%)	15/35 (42.9%)		3/7 (42.9%)	4/9 (44.4%)	
BD early	8/13 (61.5%)	5/22 (22.7%)		10/17 (58.8%)	16/35 (45.7%)		3/7 (42.9%)	4/9 (44.4%)	
MDS early	1/13 (7.7%)	4/22 (18.2%)		2/17 (11.8%)	4/35 (11.4%)		1/7 (14.3%)	1/9 (11.1%)	
Interval between the two diagnoses (yrs), median (range)	2 (0, 9)	0 (0, 14)	0.173	1 (0, 40)	2 (0, 10)	0.494	1 (0, 15)	1 (0, 10)	NA
Intestinal extent, n (%)									
Ileal	2/9 (22.2%)	4/19 (21.1%)	1	4/15 (26.7%)	11/33 (33.3%)		1/4 (25%)	2/7 (28.6%)	1
IC	5/9 (55.6%)	12/19 (63.2%)	1	2/15 (13.3%)	10/33 (30.3%)		1/4 (25%)	2/7 (28.6%)	1
Colonic	5/9 (55.6%)	4/19 (21.1%)	0.097	5/15 (33.3%)	6/33 (18.2%)		1/4 (25%)	4/7 (57.1%)	1
Whole colon	1/9 (11.1%)	3/19 (15.8%)	1	4/15 (26.7%)	8/33 (24.2%)		1/4 (25%)	NA	0.364
Rectum	NA	1/19 (5.3%)		4/15 (26.7%)	1/33 (3.0%)		NA	NA	
MDS subtype, n (%)			0.107			0.040			
low risk	12/13 (92.3%)	13/21 (61.9%)		5/17 (29.4%)	22/37 (59.5%)				
high risk	1/13 (7.7%)	8/21 (38.1%)		12/17 (70.6%)	15/37 (40.5%)				
Trisomy 8, n (%)			0.586			0.296			
Positive	9/11 (81.8%)	20/22 (90.9%)		0/10 (0%)	4/24 (16.7%)				
Negative	2/11 (18.2%)	2/22 (9.1%)		10/10 (100%)	20/24 (83.3%)				
MDS evolution, n (%)			0.328			0.487			
Yes	3/13 (23.1%)	2/23 (8.7%)		5/18 (27.8%)	6/38 (15.8%)				
No	10/13 (76.9%)	21/23 (91.3%)		13/18 (72.2%)	32/38 (84.2%)				
Follow-up (yrs), median (range)	1.5 (0, 5)	1 (0, 5)	0.841	1.7 (1, 8.5)	2 (0, 10)	0.02	1.5 (0, 6)	2 (0, 15)	NA

AA, aplastic anemia; BD, Beçhet’s disease; CD, Crohn’s disease; IBD, inflammatory bowel disease; IC, ileocecal region; MDS, myelodysplastic syndrome; UC, ulcerative colitis.

The effects of treatment on mortality in patients with BD/IBD & MDS are shown in **Supplementary Table 4**. In patients with IBD & MDS, steroids, 5-ASA and immunomodulators were the most commonly used treatments, whereas in those with BD & MDS, stem cell transplantation (SCT) was the third most frequent treatment after steroids and immunomodulators. Higher proportions of SCT and biologic agents were observed in the BD & MDS group than in the IBD & MDS group (44.1% vs 6.7%). No treatment reached statistical significance in the univariable analysis of mortality; this finding should not be interpreted as evidence of absence of treatment benefit.

### 3.4 Risk factors

Considering follow-up duration and the number of available death events, factors associated with mortality in patients with BD & MDS and IBD & MDS were evaluated using univariable Cox regression, and the findings are summarized in **Table 3**. In patients with BD & MDS, male sex and MDS evolution were associated with mortality, with hazard ratios (HRs) and 95% confidence intervals (CIs) of 8.60 (2.02-36.54) for male versus female, and 4.66 (1.10-19.79) for MDS evolution versus no MDS evolution, respectively. Additionally, in patients with IBD & MDS, those with a high-risk MDS subtype had significantly higher mortality, with an HRs (95% CIs) of 4.0 (1.35-11.84) compared to patients with a low-risk MDS subtype. In contrast, factors such as gender, IBD type, diagnosis interval, diagnosis sequence, intestinal extent of ulcers, and trisomy 8 were not significantly associated with mortality. Given the limited number of events and wide confidence intervals, these estimates should be interpreted as exploratory.

**Table 3:** Risk factors associated with mortality of patients with BD/IBD & MDS.

Variables	BD & MDS		IBD & MDS	
	HR (95%CI)	P value	HR (95%CI)	P value
Gender (male vs female)	8.60 (2.02-36.54)	0.004	0.60 (0.23-1.58)	0.300
IBD type (CD vs UC)			0.76 (0.26-2.21)	0.619
Age at BD/IBD diagnosis (per year increase)	1.01 (0.98-1.04)	0.670	1.01 (0.98-1.04)	0.435
Age at MDS diagnosis (per year increase)	1.01 (0.98-1.04)	0.518	1.03 (0.99-1.06)	0.140
Interval between two diseases diagnosis (per year increase)	1.07 (0.89-1.27)	0.481	1.02 (0.98-1.07)	0.254
Diagnosis sequence		0.792		0.605
BD/IBD early vs simultaneously	1.53 (0.45-5.17)	0.495	1.76 (0.55-5.63)	0.338
MDS early vs simultaneously	0.81 (0.07-9.52)	0.869	1.20 (0.22-6.57)	0.836
Intestinal involvement				
IC vs no IC	0.98 (0.24-3.98)	0.977		
Ileal vs no ileal			0.85 (0.30-2.42)	0.754
whole colon vs no whole colon			1.34 (0.43-4.19)	0.610
MDS subtype (high risk vs low risk)	0.36 (0.05-2.80)	0.328	4.0 (1.35-11.84)	0.012
Trisomy 8 (yes vs no)	0.25 (0.05-1.38)	0.111	0.04 (0-139.42)	0.438
MDS evolution (yes vs no)	4.66 (1.10-19.79)	0.037	1.76 (0.60-5.12)	0.300

BD, Beçhet’s disease; CD, Crohn’s disease; IBD, inflammatory bowel disease; IC, ileocecal region; MDS, myelodysplastic syndrome; UC, ulcerative colitis.

## 4 Discussion

To our knowledge, this is the largest systematic review and pooled analysis focusing on intestinal ulcers and pancytopenia. This study yielded several key observations. A pronounced female predominance and high prevalence of trisomy 8 were found in patients with BD & MDS, whereas most IBD patients with pancytopenia were male, and the majority of IBD patients with MDS lacked trisomy 8. Mortality was substantial across subgroups (31.3%–44.4%). Male sex and MDS evolution in BD & MDS, and high-risk MDS subtypes in IBD & MDS, were associated with increased mortality. Although previous case reports suggested that biologic agents (18, 50, 52) and SCT (13, 15, 20, 21, 27, 28, 54) may be useful for these patients, no treatment demonstrated a statistical significant association with reduced mortality in our univariable analyses.

Thiopurine and mesalazine have been reported to be associated with pancytopenia in IBD patients (40, 56). A previous systematic review identified many drugs that can cause pancytopenia, mainly AA (2). In our study, thiopurine was associated with MDS onset in 3 CD cases, 2 UC cases and one unclassified IBD case (53, 56, 60). Mesalazine was associated with AA onset in 3 IBD patients (40, 43, 47). Given the small numbers, larger controlled studies are needed to further clarify the roles of these drugs.

Here we report for the first time remarkable sex imbalances in these patients. 68.9% of patients with BD and MDS were female, whereas males accounted for up to 90% of IBD patients with pancytopenia. Epidemiologically, no sex predominance exists in UC or CD (67, 68), the female-to-male ratio in intestinal BD patients is around 1.2 (54.6% vs 45.4%) (69, 70), and AA occurs equally in both sexes (71); MDS shows a slight male predominance in the general population (72). In the largest case series on IBD & MDS, Harewood et al. reported a male predominance (80%), especially in CD patients (86%) (10). Thus, the observed sex imbalance may serve as a useful clinical clue in the initial evaluation of these patients.

Trisomy 8 is a common chromosomal abnormality in MDS and can be found in 6.5%-20% of patients, but the relationship between trisomy 8 and MDS pathogenesis remains unclear (31, 73). Several publications have noted trisomy 8 in MDS patients with intestinal ulcers and suggested that it may be a potential etiologic factor, but prevalence data were lacking. Here, we found a notable distribution difference: 85.7% of BD & MDS patients harbored trisomy 8, compared with only 17.9% of CD & MDS patients and none of the UC & MDS patients. Thus, trisomy 8 may serve as a preliminary clinical clue for distinguishing BD-associated and IBD-associated MDS. To date, a hypothesis to explain the high incidence of trisomy 8 in BD-associated with MDS is still lacking. Detailed analyses by comparing the role of trisomy 8 in MDS & BD with that in MDS & IBD may help generate mechanistic hypotheses regarding MDS combined with intestinal ulcers.

Previous studies noted a poor prognosis of patients with intestinal ulcers and pancytopenia, but did not define mortality rate. Here, we found strikingly high mortalities (31.3% to 44.4%) with a median follow-up of 2 years. Considering the following facts: (1) mortality from intestinal BD is low (5%-9.8% in cohort studies). In a 20-years follow-up study involving 428 BD patients, a total of 42 (cumulative mortality of 9.8%) died at the end of the survey (74). And a French cohort study of 817 patients showed a mortality rate of 5% during a median follow-up of 8 years (75); (2) UC patients do not have increased mortality compared with general population (67); (3) CD patients have a slightly higher mortality risk than general population (76); (4) The 3-year age-adjusted projected survival for MDS is about 60.0% (77); (5) Infections remain the major cause of death in AA (78), and 5-year mortality in severe AA has decreased from 36% to 21% (79). Thus, the high mortality in patients with intestinal ulcer and MDS may partly reflect MDS progression, whereas patients with IBD & AA likely experience higher reported mortality than general IBD patients and AA patients.

Although previous case reports described successful treatment of IBD/BD and MDS with biological agents such as adalimumab and ustekinumab (18, 50, 52) or SCT (13, 15, 20, 21, 27, 28, 54), neither biologic agents nor SCT reached statistical significance in the univariable mortality analysis. Possible explanations include: 1) biologic agents may be less effective in some patients or may increase infection risk, which can be more severe in patients with pancytopenia due to the leukopenia (8, 17). Consequently, the survival potential of these patients may be affected. In addition, theoretically, allogeneic SCT is the only therapeutic measure that offers a potential cure for MDS and SCT was also reported to have a capability to remove trisomy 8, which is likely to be an etiologic factor for intestinal ulcer combined with MDS (13, 80, 81). However, the immunosuppressive effect from intensity of chemotherapy in “conditioning” process, which could potentially induce several major complications such as mucositis, bleeding and infections may also affect the outcomes, considering the leukopenia nature of these patients. The SCT-related mortality and relapse rate after SCT were reported as 15% to 30% and 30% to 50%, respectively (82, 83); 2) Because the sample sizes in the subgroups were small, the possibility that statistical power was not enough to detect significant differences should not be excluded. In addition, treatment selection was heterogeneous and may have been confounded by indication, disease severity, and treatment timing.

Regarding factors associated with mortality, male sex and MDS evolution in BD & MDS and high-risk MDS subtype in IBD & MDS were associated with mortality in univariable analyses. Especially for male, the risk of mortality in a median follow-up of 2 years is 8 times that of females in patients with BD & MDS. Similar findings have also been reported that in a French study, male to female mortality ratio in general BD patients was as high as 5 times (75). MDS subtype has previously been identified as an independent risk factor for mortality (84, 85). These hazard ratios are unadjusted and should be interpreted as providing clues for further investigation, although accumulating larger cohorts (particularly prospective ones) with sufficient events to identify true confounders is challenging. Given the limited number of deaths, these findings should not be interpreted as independent prognostic predictors.

From a clinical perspective, these findings may have implications for diagnosis, prognosis, and management. In patients with atypical or refractory intestinal ulcers accompanied by unexplained cytopenia, early review of drug exposure, serial blood counts, bone marrow examination, and cytogenetic testing—particularly assessment for trisomy 8 when intestinal BD is suspected—may provide preliminary clues for considering MDS/AA-associated disease rather than conventional IBD or BD. Prognostically, male sex, MDS evolution, and higher-risk MDS subtypes should be regarded as exploratory prognostic signals rather than definitive risk markers. Therapeutically, multidisciplinary care involving gastroenterologists and hematologists is likely essential, and future prospective studies are needed to clarify the roles of biologic therapy, hypomethylating agents, and hematopoietic stem-cell transplantation in this rare but high-risk population.

The main strength of this study is the systematic, comprehensive literature search of all available cases, individual patient data analysis using a predefined protocol, and use of recommended approach for methodological assessment (12). Several additional limitations should be considered. First, small subgroup sample size, especially for IBD & AA and BD & AA, is the main limitation. Due to inadequate numbers of target events, multivariable Cox regression cannot be performed; instead, the risk factors found in this study should be interpreted as clues to guide further studies. Given the rarity of these cases, this study represents the largest sample to date and is likely to remain so for the foreseeable future. In addition, the evidence base was dominated by case reports and small case series, with unavoidable heterogeneity, potential publication bias, and limited generalizability; nevertheless, pooling published case-level data is currently the most feasible strategy for summarizing this rare condition. Adverse events were missing in several included publication, thus, outcomes associated with adverse events cannot be analyzed. Missing data were handled using available-case analysis without imputation, and restriction to English-language full texts may have introduced language bias. Therefore, the findings should be interpreted as descriptive and hypothesis-generating rather than definitive.

## 5 Conclusion

Intestinal ulceration with concurrent pancytopenia is rare but associated with substantial short-term mortality, warranting heightened clinical vigilance. The observed sex distribution and trisomy 8 patterns should be viewed as hypothesis-generating observations rather than definitive evidence of distinct pathobiology or diagnostic utility. None of the commonly used treatment, such as corticosteroids, immunomodulators, 5-aminosalicylic acid, biologic agents, or hematopoietic stem-cell transplantation, showed a statistically significant survival benefit in univariable analyses, underscoring the need for improved, evidence-based treatment strategies. Male sex, MDS progression, and high-risk MDS subtypes were exploratory mortality-associated signals that require confirmation in larger datasets. Future work should focus on prospective registries and mechanistic studies to improve management of this rare but high-risk entity.

## Data Availability

The raw data supporting the conclusions of this article will be made available by the authors, without undue reservation.
