# Supplementary material for: When intestinal ulceration meets hematologic malignancies: clinical features and mortality from a pooled individual-patient data systematic review
Source: Front Immunol. 2026 May 29;17:1808470. doi: 10.3389/fimmu.2026.1808470 (PMC13260510; doi:10.3389/fimmu.2026.1808470)
Supplement: Supplementary file 4 [file SupplementaryFile1.docx]

**Clinical characteristics and mortality of IBD or intestinal Behçet’s with concurrent pancytopenia: a systematic review and pooled individual patient data analysis**

Song Su ^1 #^, He Zhou ^1 #^, Zimeng Wang ^1^, Yanting Shi ^2^, Xiaofei Li ^3^, Longsong Li ^1^, Yawei Bi ^1^, Jie Liang ^2 *^, Ningli Chai ^1 *^

^1^ Senior Department of Gastroenterology ,Chinese PLA General Hospital, Beijing, China, 100039.

^2^ State key Laboratory of Cancer Biology and National Clinical Research Center for Digestive Diseases, Xijing Hospital of Digestive Diseases, Fourth Military Medical University (Air Force Medical University), Xi’an, China, 710032.

^3^ Second Division of Cadre Ward, General Hospital of Central Theater Command, Wuhan, 430000.

**Correspondence:**

Prof. Ningli Chai, E-mail: chainingli@vip.163.com

Prof. Jie Liang, E-mail: [liangjie@fmmu.edu.cn](mailto:liangjie@fmmu.edu.cn)

This file includes:

Supplemental Fig. 1-3

Supplemental Table 1-4


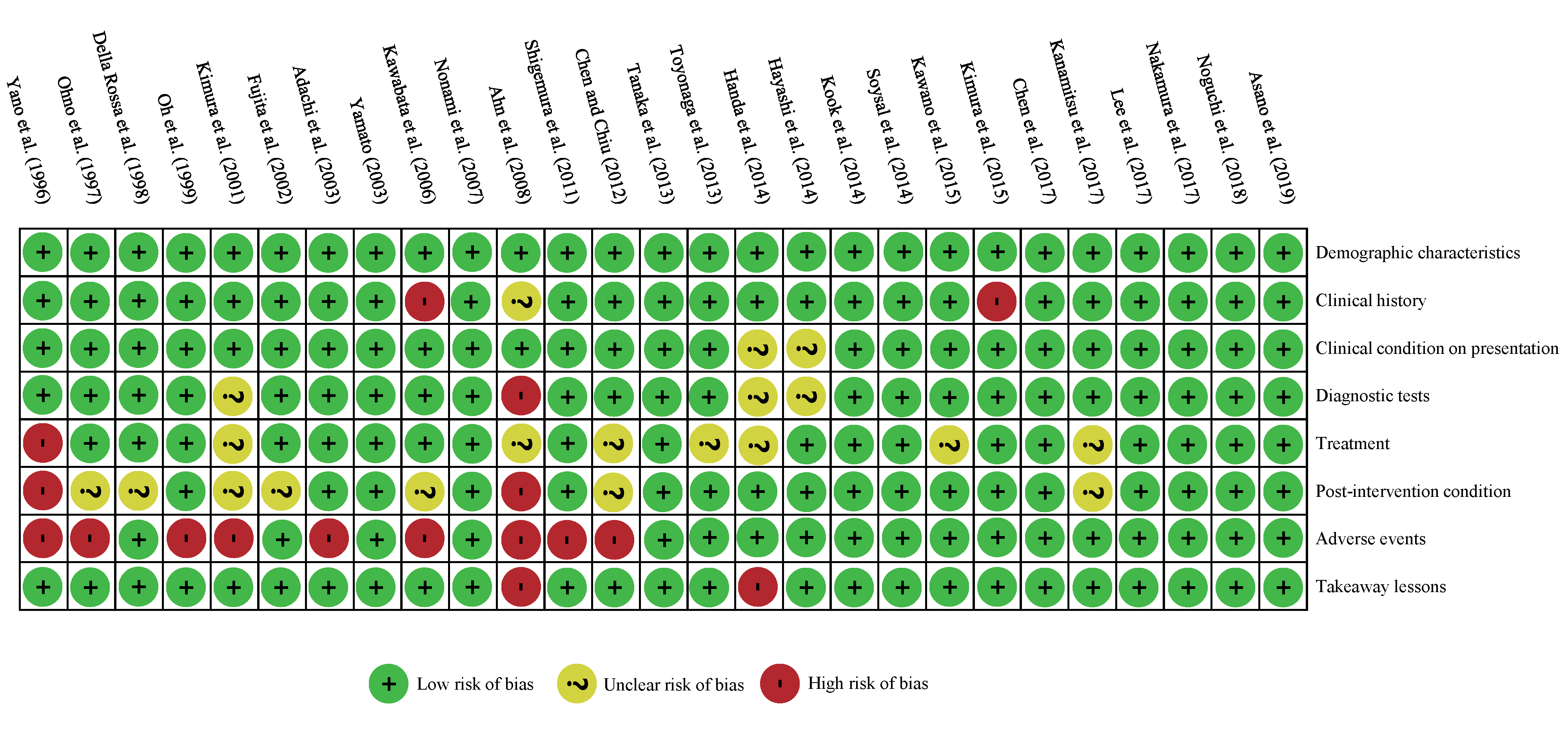


Supplemental Figure 1: Methodological quality of studies on BD and MDS.


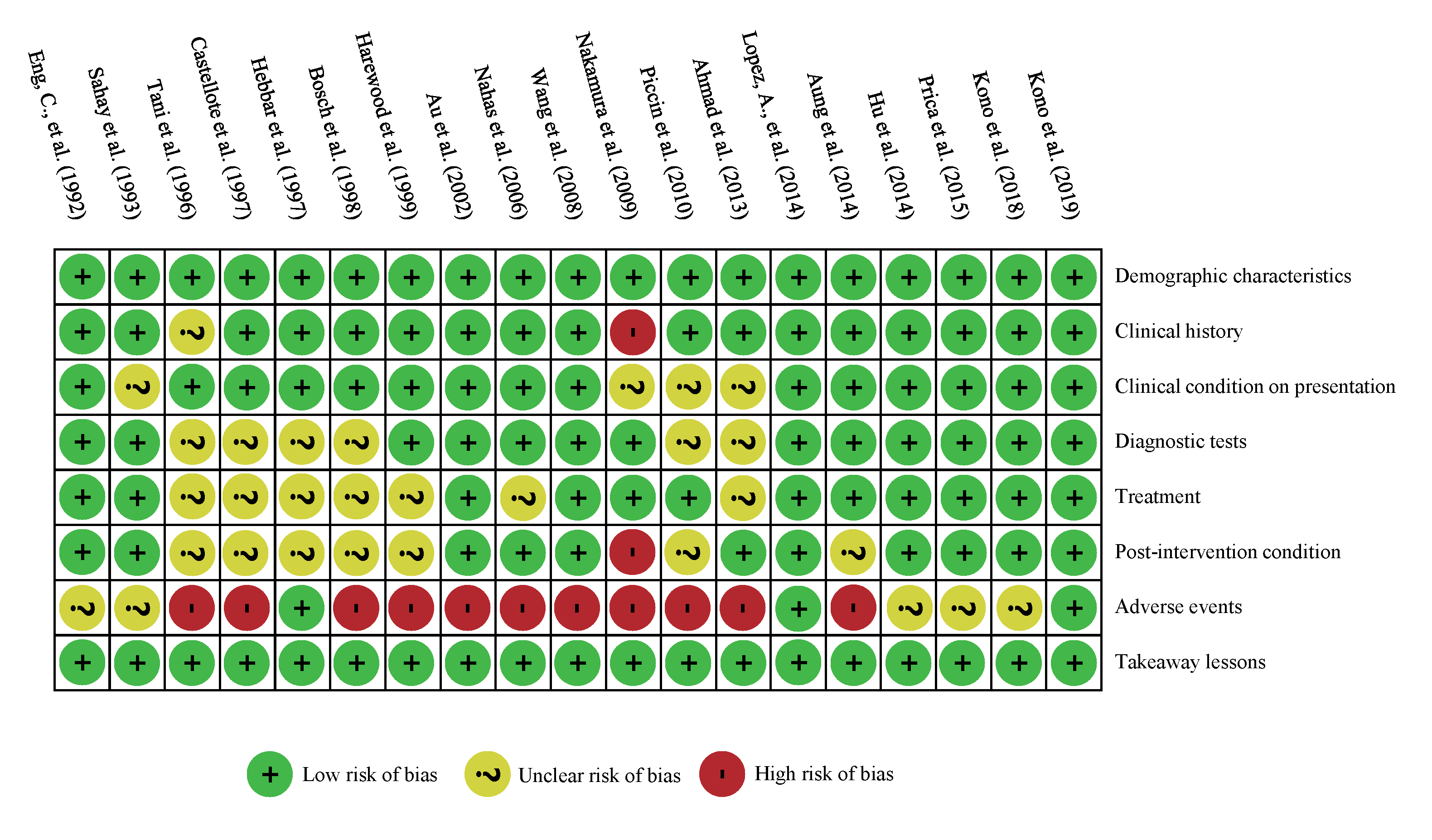


Supplemental Figure 2: Methodological quality of studies on IBD and MDS.


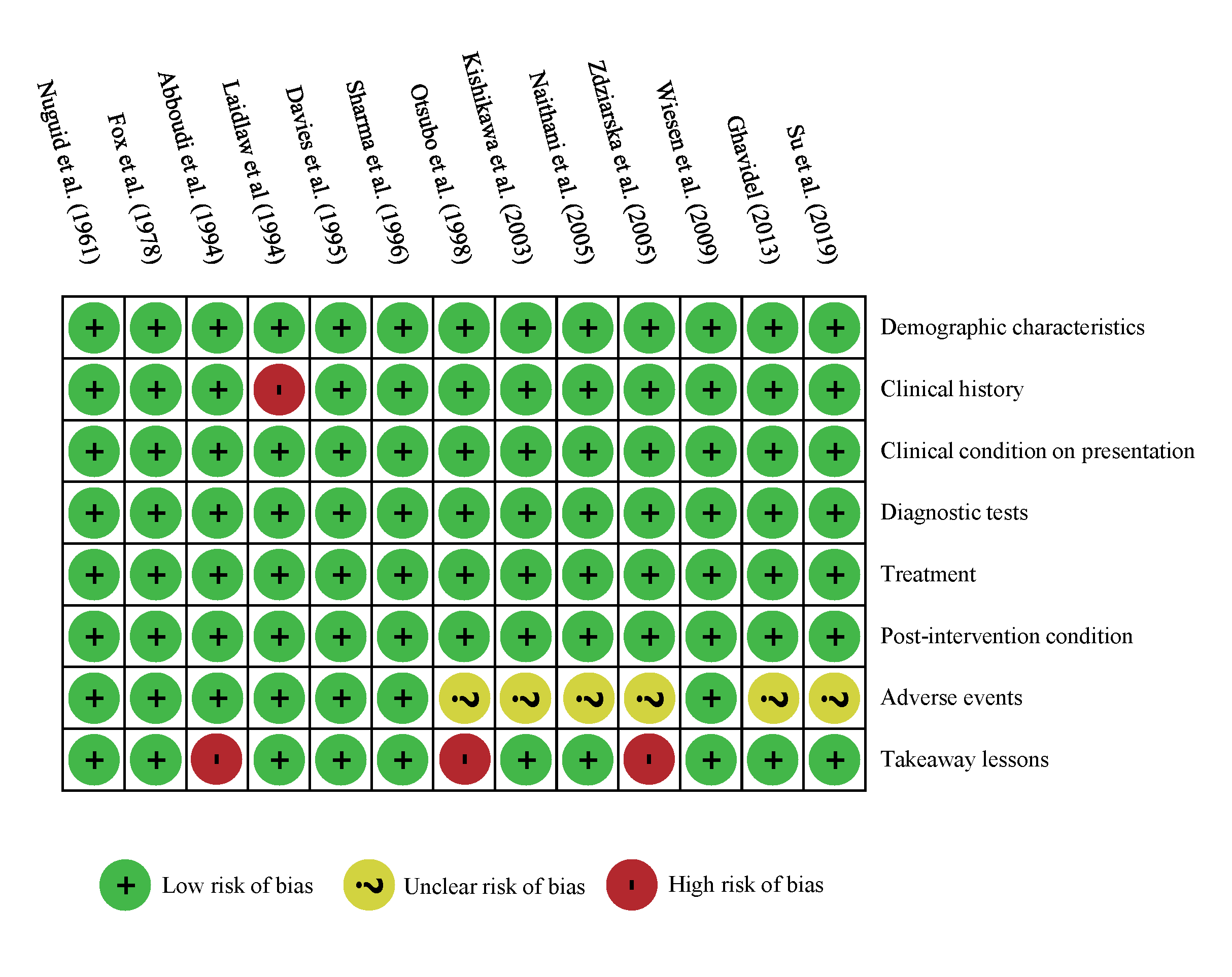


Supplemental Figure 3: Methodological quality of studies on IBD and AA.

**Supplemental Table 1: characteristics of studies on BD and MDS**

| **Study** | **Country** | **N** | **Gender** | **Age at BD diagnosis,  yrs** | **BD extent** | **Age at MDS diagnosis,  yrs** | **MDS subtype** | **Trisomy 8** | **Treatment** | **MDS evolution** | **Follow-up, yrs** | **Vital status** |  |
| --- | --- | --- | --- | --- | --- | --- | --- | --- | --- | --- | --- | --- | --- |
| **Asano, T., et al. (2019)** | Japan | 1 | F | 11 | IC | 18 | RCMD | + | steroid; surgery; SCT; | None | 0.7 | Alive |  |
| **Noguchi, M., et al. (2018)** | Japan | 1 | M | 5 | NS | 7 | RCMD | - | chemotherapy; steroid; SCT; immunomodulator; G-CSF; biologic | AML | 0.25 | Dead |  |
| **Lee, W. S., et al. (2017)** | Korea | 1 | F | 54 | IC | 52 | RCMD | + | steroid; immunomodulator; 5-ASA; chemotherapy; SCT; G-CSF | None | 2 | Alive |  |
| **Kanamitsu, K., et al. (2017)** | Japan | 4 | 4 F | 4; 8; 10; 4 | 1 IC+Colonic; 3 NS | 7; 11; 10; 4 | 2 RAEB-t; 1 RA; 1 RAEB | 2+; 2- | 4/4 SCT; 3/4 Chemotherapy; 2/4 immunomodulator; | 1 RAEB; 1 AML; 2 None | 4; 2; 2 NS | 2 Dead; 1 Alive |  |
| **Chen, Y., et al. (2017)** | China | 1 | F | 46 | IC | 46 | NS | + | steroid; immunomodulator; biologic; G-CSF; | None | 1 | Alive |  |
| **Kimura, M., et al. (2015)** | Japan | 1 | F | 79 | IC | 79 | RAEB-1 | + | biologic; | None | 0.4 | Alive |  |
| **Kawano, S., et al. (2015)** | Japan | 3 | 3 M | 81; 40; 64 | 3/3 IC; 1/3 Ileal; 1/3 Colonic | 81; 45; 62 | 1 RARS; 2 RA | 3+ | 2/3 SCT; 1/3 immunomodulator; 1/3 surgery; | 3 None | 0.7; 0.25; NS | 3 Dead |  |
| **Soysal, T., et al. (2014)** | Turkey | 1 | F | 25 | Colonic; Rectum | 30 | RAEB-2 | + | SCT | None | 1.7 | Alive |  |
| **Kook, M. H., et al. (2014)** | Korea | 1 | F | 38 | IC | 42 | RCMD | + | chemotherapy; SCT; immunomodulator; | None | 5 | Alive |  |
| **Hayashi, K., et al. (2014)** | Japan | 1 | M | NS | NS | NS | RAEB-1 | NS | chemotherapy; immunomodulator; ATG; steroids; SCT | None | 0.3 | Alive |  |
| **Handa, T., et al. (2014)** | Japan | 2 | 1 F; 1 M | 37; 27 | 2 NS | 46; 32 | 2 RA | 2 NS | 1 steroid+ immunomodulator+ biologic; 1 NS | 1 AML; 1 None | 0.5; 2 | 2 Dead |  |
| **Toyonaga, T., et al. (2013)** | Japan | 2 | 1 M; 1 F | 36; 29 | 1/2 IC; 1/2 Colonic | 36; 37 | 2 RA | 2 + | 2/2 5-ASA; 2/2 steroid; 2/2 immunomodulator; 2/2 biologic | 2 None | 3; NS | 1 Dead; 1Alive |  |
| **Tanaka, H., et al. (2013)** | Japan | 1 | M | 59 | IC; Whole colon | 59 | RCMD | + | 5-ASA; steroid; immunomodulator; biologic; chemotherapy | None | NS | Alive |  |
| **Chen, H. C. and Y. M. Chiu (2012)** | China | 1 | F | 24 | Ileal; Colonic | 24 | RA | + | NS | None | NS | Alive |  |
| **Shigemura, T., et al. (2011)** | Japan | 1 | F | 4 | Colonic | 4 | NS | + | SCT | None | 3 | Alive |  |
| **Ahn, J. K., et al. (2008)** | Korea | 6 | 5 F; 1 M | 46.5 (12.8) | 6 NS | 47.7 (12.0) | 1 RA; 3 RCMD; 1 RARS; 1 MDS-U | 4+; 2- | 1 SCT; 5 NS | 6 None | 6 NS | 6 NS |  |
| **Nonami, A., et al. (2007)** | Japan | 1 | F | 28 | Whole colon | 27 | RAEB | + | G-CSF; SCT | None | 2 | Alive |  |
| **Kawabata, H., et al. (2006)** | Japan | 2 | 1 F; 1 M | 80; 75 | 2/2 IC | 76; 75 | 1 RA; 1 RAEB | 2+ | 2/2 5-ASA; | 2 None | 1; 1 NS | 2 Alive |  |
| **Yamato, K. (2003)** | Japan | 1 | F | 10 | Ileal; Colonic | 10 | RAEB | - | steroids; chemotherapy; SCT; immunomodulator; | AML | 1 | Alive |  |
| **Adachi, Y., et al. (2003)** | Japan | 2 | 2 F | 28; 23 | 2/2 IC | 28; 37 | 2 RA | 2 + | 2/2 steroid; 2/2 5-ASA; 2/2 surgery; | 2 None | 2; 0.7 | 2 Alive |  |
| **Fujita, H., et al. (2002)** | Japan | 1 | M | 64 | Ileal; Whole colon | 64 | RA | + | steroid; surgery | None | 2 | Alive |  |
| **Kimura, S., et al. (2001)** | Japan | 3 | 3 F | 74; 36; 31 | 3/3 Colonic; 1/3 Ileal | 74; 36; 32 | 3 RA | 3+ | 2/2 steroids; 1/2 G-CSF; 1 NS | 3 None | 1; 5; 4 | 3 Dead |  |
| **Oh, E. J., et al. (1999)** | Korea | 1 | F | 50 | IC | 50 | RA | + | steroid | None | 2 | Alive |  |
| **Della Rossa, A., et al. (1998)** | Italy | 1 | M | 50 | IC | 50 | RARS | + | steroids; chemotherpay | None | 0.1 | Dead |  |
| **Ohno, E., et al. (1997)** | Japan | 1 | F | 37 | NS | 34 | RA | + | NS | None | 0.75 | Alive |  |
| **Yano, K., et al. (1996)** | Japan | 1 | F | 19 | IC | 23 | RA | + | NS | None | 0 | Alive |  |
| **Ogawa, H., et al. (2001)** | Japan | 2 | 1 M; 1 F | 30; 41 | 2 NS | 25; 41 | 2 RA | 2+ | 2 NS | 2 None | 2 NS | 2 NS |  |
| **Karuvannur, S., et al. (2001)** | Japan | 1 | M | 67 | NS | NS | RA | + | NS | None | NS | NS |  |

BD, Bechet’s disease; MDS, myelodysplastic syndrome; IC, ileocecal region; AML, acute myeloid leukemia; RAEB, refractory anemia with excess of blasts; CML, chronic myelomonocytic leukemia; RAEB-t: refractory anemia with excess blasts in transformation; RCMD, refractory cytopenia with multilineage dysplasia; RARS, refractory anemia with ringed sideroblasts; G-CSF, granulocyte colony-stimulating factor; SCT, stem cell transplantation; 5-ASA, 5-aminosalicylic acid; ATG, anti-thymocyte globulin;

**Supplemental Table 2: characteristics of studies on IBD and MDS**

| **Study** | **Country** | **N** | **Gender** | **Age at IBD diagnosis, yrs** | **IBD type** | **IBD extent** | **Age at MDS diagnosis, yrs** | **MDS**  **subtype** | **Trisomy 8** | **Treatment** | **MDS evolution** | **Follow-up, yrs/median (range)** | **Vital status** |
| --- | --- | --- | --- | --- | --- | --- | --- | --- | --- | --- | --- | --- | --- |
| **Kono, M., et al. ( 2019)** | Japan | 1 | M | 69 | CD | IC | 63 | RCMD | + | biologics; chemotherapy; | None | 1.3 | Alive |
| **Kono, M., et al. ( 2018)** | Japan | 1 | M | 75 | IBD-U | IC; Whole colon | 70 | RAEB-2 | - | 5-ASA; immunomodulator; | None | 0.5 | Alive |
| **Prica, A., et al. (2015)** | Canada | 1 | M | NS | CD | NS | 60 | RCMD | - | biologics; chemotherapy; | None | 6 | Alive |
| **Lopez, A., et al. ( 2014)** | France | 3 | 2 M,1 F | NS | 2 CD, 1 IBD-U | 1/2 Ileal; 1/2 IC; 1 NS | 49, 53, 81 | 3 RAEB-1 | 3 NS | 3 NS | 3 None | 3 NS | 2 Dead; 1 Alive; |
| **Hu, C., et al. (2014)** | China | 1 | M | 50 | CD | Ileal; Colonic | 50 | RAEB-1 | + | steroid; immunomodulator; chemotherapy; SCT; ATG; | None | 2 | Alive |
| **Aung, P. P., et al. (2014)** | USA | 1 | F | NS | UC | NS | 67 | RAEB-2 | NS | steroid; chemotherapy; 5-ASA | AML | 0.5 | Alive |
| **Ahmad, O. F., et al. ( 2013)** | UK | 2 | 1F, 1M | 62, 18 | 2 UC | 2 NS | 68, 22 | 1 RAEB-2, 1 CML | 2 - | 1/2 biologics; 1/2 5-ASA; 1/2 steroid; 2/2 chemotherapy; 1/2 operation; | 2 None | 0.7; 0.7 | 2 Dead |
| **Piccin, A., et al. (2010)** | Italy | 1 | M | 8 | CD | NS | 14 | NS | - | SCT | AML | 6 | Alive |
| **Nakamura, F., et al. ( 2009)** | Japan | 1 | M | 55 | IBD-U | Colonic | 55 | NS | + | immunomodulator | None | NS | Alive |
| **Wang, Z., et al. ( 2008)** | China | 9 | 2 F, 7 M | 38.7 (10.4 | 2 UC, 7 CD | 5/9 Ileal; 2/9 Whole colon; 2/9 Colonic; 1/9 Rectum | 38.3 (9.7 | 3 RA, 3 RAEB, 2 RAEB-T, 1 NS | 9 NS | 7/9 steroid, 4/9 5-ASA, 4/9 retinoic acid, 3/9 immunomodulator,1/9 chemotherapy; | 9 None | 1.25 (0.2-8.5) | 2 Dead; 7 Alive |
| **Nahas, S. C., et al. (2006)** | Brazil | 1 | M | 9 | CD | IC | 9 | RAEB-2 | + | steroid; 5-ASA; operation; G-CSF; chemotherapy | AML | 2 | Alive |
| **Au, W. Y., et al. ( 2002)** | China | 1 | M | 42 | CD | NS | 48 | t-MDS | + | NS | None | 1 | Alive |
| **Harewood, G. C., et al. ( 1999)** | USA | 25 | 8 F; 17 M | 55.2 (16.3 | 14 CD; 11 UC | 9/25 Whole colon; 8/25 Ileal; 4/25 IC; 3/25 Colonic; 1/25 Rectum; | 66 (12.4 | 11 RA; 7 RARS; 5 RAEB; 2 CML | 20 -; 5 NS | 25 NS | 3 AML; 22 None | 1 (0-7) | 7 Dead; 18 Alive |
| **Bosch, X., et al. (1998)** | British | 3 | 2 M; 1 F | 82; 68; 72 | 3 CD | 1/3 IC+Colonic; 2NS | 82; 68; 72 | 2 RAEB; 1 RAEB-T | 2 -; 1 NS | 3/3 steroid; 3/3 5-ASA | 1 AML; 2 None | 0.5; 2; 1.3 | 1 Dead; 2 Alive |
| **Hebbar, M., et al. (1997)** | Belgium | 6 | 3 M; 3 F | 59 (14.3 | 6 CD | 3/6 IC; 3/6 Rectum; 3/6 Colonic | 61.7 (15 | 3 RA; 1 RARS; 1 RAEB; 1 RAEB-t | 3 -; 3 NS | 4/5 steroid; 2/5 5-ASA; 1/5 Chemotherapy; 1 NS; | 2 CML;  1 RAEB-T; 3 None; | 2.5 (0.1-10) | 3 Dead; 3 Alive |
| **Castellote, J., et al. (1997)** | Spain | 1 | M | 52 | CD | Colonic | 82 | RARS | NS | steroid | None | NS | NS |
| **Tani, T., et al. (1996)** | Japan | 1 | M | 28 | CD | IC; Whole colon | 28 | RAEB-T | - | 5-ASA; steroid; Chemotherapy; Immunomodulator; | AML | 2 | Dead |
| **Sahay, R., et al. ( 1993)** | USA | 2 | 2 M | 71; 44 | 2 CD | 1 Colonic; 1 NS | 72; 66 | 1 RAEB; 1 RA | 1 -; 1 NS | 1 5-ASA; 1 NS | 2 None | 1 0.5; 1 NS | 1 Dead; 1 NS |
| **Eng, C., et al. (1992)** | USA | 4 | 3 F; 1 M | 87; 78; 56; 68 | 4 CD | 1 Whole colon; 3 NS | 83; 78; 56; 68 | 3 RARS; 1 RA | 3 -; 1 + | 2/3 5-ASA; 1/3 steroid; 1 NS | 1 AML; 3 None | 4 NS | 4 NS |

IBD, inflammatory bowel disease; MDS, myelodysplastic syndrome; CD, Crohn’s disease; UC, ulcerative colitis; IBD-U, IBD undetermined; IC, ileocecal region; AML, acute myeloid leukemia; RAEB, refractory anemia with excess of blasts; CML, chronic myelomonocytic leukemia; RAEB-t: refractory anemia with excess blasts in transformation; RCMD, refractory cytopenia with multilineage dysplasia; RARS, refractory anemia with ringed sideroblasts; t-MDS, treatment-related myelodysplastic syndrome; SCT, stem cell transplantation; G-CSF, granulocyte colony-stimulating factor; 5-ASA, 5-aminosalicylic acid; ATG, anti-thymocyte globulin;

**Supplemental Table 3: characteristics of studies on IBD and AA**

| **Study** | **Country** | **N** | **Gender** | **Age at IBD diagnosis, yrs** | **IBD type** | **IBD extent** | **Age at AA diagnosis, yrs** | **Treatment** | **Follow-up, yrs/median (range)** | **Vital status** |
| --- | --- | --- | --- | --- | --- | --- | --- | --- | --- | --- |
| **Su, S., et al. (2019)** | China | 6 | 6 M | 41.5 (20.1) | 6 CD | 3/6 Colonic; 2/6 IC; 2/6 Ileal; 1/6 Whole colon | 39.2 (22.7) | 5/6 Mesalazine; 4/6 steroid; 4/6 immunomodulator; 2/6 surgery; 1/6 G-CSF; 1/6 biologic | 2.5 (0.9,8) | 4 Dead; 2 Alive |
| **Ghavidel, A. (2013)** | Iran | 1 | M | 38 | UC | Colonic | 43 | steroid; immunomodulator | 2 | Alive |
| **Wiesen, A., et al. (2009)** | USA | 1 | F | NS | CD | IC | 52 | immunomodulator; steroid | 1 | Alive |
| **Zdziarska, B., et al. (2005)** | Poland | 1 | M | 45 | CD | NS | 49 | steroid | 0.1 | Alive |
| **Naithani, R., et al. (2005)** | India | 1 | M | 41 | CD | IC; Colonic | 31 | immunomodulator; steroids | NS | NS |
| **Kishikawa, H., et al. (2003)** | Japan | 1 | M | 28 | UC | Colonic | 27 | mesalazine; steroid; immunomodulator | 4 | Alive |
| **Otsubo, H., et al. (1998)** | Japan | 1 | M | 20 | UC | NS | 20 | mesalazine; immunomodulator;  G-CSF | NS | Alive |
| **Sharma, B. C., et al. (1996)** | India | 1 | M | 9 | UC | Colonic | 9 | steroid; mesalazine | 1.5 | Alive |
| **Davies, J. M., et al. (1995)** | USA | 1 | M | 43 | CD | Ileal | 53 | steroid; immunomodulator; surgery | 15 | Alive |
| **Laidlaw, S. T., et al (1994)** | UK | 1 | M | 46 | UC | NS | 47 | steroid;immunomodulator | 1 | Alive |
| **Abboudi, Z. H., et al. (1994)** | UK | 1 | M | 71 | UC | NS | 72 | immunomodulator | 0.1 | Dead |
| **Fox, R. M., et al. (1978)** | Australia | 1 | F | 22 | UC | NS | 37 | steroid; immunomodulator | 1 | Dead |
| **Nuguid, T. P., et al. (1961)** | USA | 1 | M | 18 | UC | NS | 19 | steroid | NS | Dead |

IBD, inflammatory bowel disease; AA, aplastic anemia; CD, Crohn’s disease; UC, ulcerative colitis; IC, ileocecal region; G-CSF, granulocyte colony-stimulating factor; 5-ASA, 5-aminosalicylic acid;

**Supplemental Table 4: Relationship between treatment and mortality of patients with BD/IBD & MDS**

| **Treatment** | **BD & MDS** | | | | | **IBD & MDS** | | | | |
| --- | --- | --- | --- | --- | --- | --- | --- | --- | --- | --- |
|  | **Total**  **(n=34)** | **Dead**  **(n=13)** | **Alive**  **(n=21)** | **HR (95%CI) *** | **p value** | **Total**  **(n=30)** | **Dead**  **(n=11)** | **Alive**  **(n=19)** | **HR (95%CI) *** | **p value** |
| **Steroids** | 26 (76.5%) | 10 (76.9%) | 16 (76.2%) | 0.85 (0.18-4.11) | 0.840 | 24 (80%) | 8 (72.7%) | 16 (84.2%) | 0.68 (0.14-3.30) | 0.630 |
| **Immunomodulators** | 17 (50%) | 7 (53.8%) | 10 (47.6%) | 1.35 (0.43-4.27) | 0.608 | 11 (36.7%) | 5 (45.5%) | 6 (31.6%) | 0.80 (0.22-2.87) | 0.734 |
| **5-ASA** | 14 (41.2%) | 3 (23.1%) | 11 (52.4%) | 0.43 (0.12-1.61) | 0.211 | 18 (60%) | 7 (63.6%) | 11 (57.9%) | 2.53 (0.52-12.20) | 0.249 |
| **SCT** | 15 (44.1%) | 5 (38.5%) | 10 (47.6%) | 0.58 (0.17-1.93) | 0.374 | 2 (6.7%) | 0 (0%) | 2 (10.5%) | 0.04 (0-514.46) | 0.508 |
| **Biological agents** | 10 (29.4%) | 4 (30.8%) | 6 (28.6%) | 2.65 (0.70-10.07) | 0.153 | 3 (10%) | 0 (0%) | 3 (15.8%) | 0.04 (0-152.72) | 0.443 |
| **surgery** | 5 (14.7%) | 1 (7.7%) | 4 (19.0%) | 0.72 (0.09-5.85) | 0.755 | 2 (6.6%) | 1 (9.1%) | 1 (5.3%) | 1.33 (0.17-10.65) | 0.773 |
| **chemotherapy** | 8 (23.5%) | 2 (15.4%) | 6 (28.6%) | 0.74 (0.16-3.49) | 0.698 | 8 (26.7%) | 4 (36.4%) | 4 (21.1%) | 1.98 (0.53-7.39) | 0.310 |

* vs no use of treatment;

BD, Bechet’s disease; IBD, inflammatory bowel disease; MDS, myelodysplastic syndrome; SCT, stem cell transplantation; 5-ASA, 5-aminosalicylic acid.
