# Supplementary material for: When intestinal ulceration meets hematologic malignancies: clinical features and mortality from a pooled individual-patient data systematic review
Source: Front Immunol. 2026 May 29;17:1808470. doi: 10.3389/fimmu.2026.1808470 (PMC13260510; doi:10.3389/fimmu.2026.1808470)
Supplement: Supplementary file 5 [file SupplementaryFile2.docx]

**IBD/BD combined with MDS/AA Search Strategy- Jan. 2020 (1.20)**

**PubMed**

**#1** (((((((((((((((((((((Triple-Symptom Complex[Title/Abstract]) OR Behcet Disease[Title/Abstract]) OR Triple Symptom Complex[Title/Abstract]) OR Complex, Triple Symptom[Title/Abstract]) OR Complices, Triple Symptom[Title/Abstract]) OR Symptom Complex, Triple[Title/Abstract]) OR Symptom Complices, Triple[Title/Abstract]) OR Triple Symptom Complices[Title/Abstract]) OR Adamantiades-Behcet Disease[Title/Abstract]) OR Adamantiades Behcet Disease[Title/Abstract]) OR Adamantiades-Behcet Diseases[Title/Abstract]) OR Disease, Adamantiades-Behcet[Title/Abstract]) OR Diseases, Adamantiades-Behcet[Title/Abstract]) OR Behcet Triple Symptom Complex[Title/Abstract]) OR Old Silk Route Disease[Title/Abstract]) OR Behcet's Syndrome[Title/Abstract]) OR Behcets Syndrome[Title/Abstract]) OR Behçet Disease[Title/Abstract]) OR Behçet Diseases[Title/Abstract]) OR Disease, Behçet[Title/Abstract]) OR Diseases, Behçet[Title/Abstract]) OR "Behcet Syndrome"[Mesh]

**#2** (((((((Inflammatory Bowel Disease[Title/Abstract]) OR Bowel Diseases, Inflammatory[Title/Abstract]) OR Crohn*[Title/Abstract]) OR Ulcerative Colitis[Title/Abstract]) OR IBD[Title/Abstract]) OR CD[Title/Abstract]) OR UC[Title/Abstract]) OR ("Inflammatory Bowel Diseases"[Mesh] OR "Crohn Disease"[Mesh] OR "Colitis, Ulcerative"[Mesh])

**#3** ((((((((Aplastic Anemias[Title/Abstract]) OR Aplastic Anemia[Title/Abstract]) OR Aplastic Anaemia[Title/Abstract]) OR Anaemia, Aplastic[Title/Abstract]) OR Aplastic Anaemias[Title/Abstract]) OR Anemia, Hypoplastic[Title/Abstract]) OR Hypoplastic Anemia[Title/Abstract]) OR Hypoplastic Anemias[Title/Abstract]) OR ("Anemia, Aplastic"[Mesh] OR "Aplastic anemia, idiopathic" [Supplementary Concept])

**#4** ((((((((((((Myelodysplastic Syndrome[Title/Abstract]) OR Syndrome, Myelodysplastic[Title/Abstract]) OR Syndromes, Myelodysplastic[Title/Abstract]) OR Dysmyelopoietic Syndromes[Title/Abstract]) OR Dysmyelopoietic Syndrome[Title/Abstract]) OR Syndrome, Dysmyelopoietic[Title/Abstract]) OR Syndromes, Dysmyelopoietic[Title/Abstract]) OR Hematopoetic Myelodysplasia[Title/Abstract]) OR Hematopoetic Myelodysplasias[Title/Abstract]) OR Myelodysplasia, Hematopoetic[Title/Abstract]) OR Myelodysplasias, Hematopoetic[Title/Abstract]) OR "Myelodysplastic Syndromes"[Mesh])

**#5** #1 OR #2

**#6** #3 OR #4

**#7** #5 AND #6

**EMBASE**

**#1** 'triple-symptom complex':ab,ti

**#2** 'behcet disease':ab,ti

**#3** 'triple symptom complex':ab,ti

**#4** 'behcet triple symptom complex':ab,ti

**#5** 'old silk route disease':ab,ti

**#6** 'behcet* syndrome':ab,ti

**#7** 'behçet disease*':ab,ti

**#8** 'behcet syndrome*':ab,ti

**#9** 'behcet disease'/mj

**#10** #1 OR #2 OR #3 OR #4 OR #5 OR #6 OR #7 OR #8 OR #9

**#11** 'inflammatory bowel disease'/mj

**#12** 'inflammatory bowel disease*':ab,ti

**#13** 'crohn*':ab,ti

**#14** 'ulcerative colitis':ab,ti

**#15** 'IBD':ab,ti

**#16** #11 OR #12 OR #13 OR #14 OR #15

**#17** #10 OR #16

**#18** 'aplastic anemia'/mj

**#19** 'aplastic anemia*':ab,ti

**#20** 'aplastic anaemia*':ab,ti

**#21** 'hypoplastic anemia*':ab,ti

**#22** #18 OR #19 OR #20 OR #21

**#23** 'myelodysplastic syndrome'/mj

**#24** 'myelodysplas*':ab,ti

**#25** 'dysmyelopoietic syndrome*':ab,ti

**#26** 'hematopoetic myelodysplasia*':ab,ti

**#27** #23 OR #24 OR #25 OR #26

**#28** #22 OR #27

**#29** #17 AND #28
